# Characterization of the Type I Restriction Modification System Broadly Conserved among Group A Streptococci

**DOI:** 10.1128/mSphere.00799-21

**Published:** 2021-11-17

**Authors:** Sruti DebRoy, William C. Shropshire, Chau Nguyen Tran, Haiping Hao, Marc Gohel, Jessica Galloway-Peña, Blake Hanson, Anthony R. Flores, Samuel A. Shelburne

**Affiliations:** a Department of Infectious Diseases Infection Control and Employee Health, University of Texas MD Anderson Cancer Center, Houston, Texas, USA; b Department of Genomic Medicine, University of Texas MD Anderson Cancer Center, Houston, Texas, USA; c JHMI Transcriptomics and Deep Sequencing Core, Johns Hopkins School of Medicine, Baltimore, Maryland, USA; d Center for Infectious Diseases, Department of Epidemiology, Human Genetics and Environmental Sciences, UTHealth School of Public Health at Houston, University of Texas Health Science Center McGovern Medical School, Houston, Texas, USA; e Division of Infectious Diseases, Department of Pediatrics, University of Texas Health Science Center McGovern Medical School, Houston, Texas, USA; f Center for Antimicrobial Resistance and Microbial Genomics, University of Texas Health Science Center McGovern Medical School, Houston, Texas, USA; g Department of Veterinary Pathobiology, Texas A&M University, College Station, Texas, USA; h Interdisciplinary Program in Genetics, College of Veterinary Medicine and Biomedical Sciences, Texas A&M University, College Station, Texas, USA; University of Nebraska Medical Center

**Keywords:** *Streptococcus pyogenes*, type I RM system, immunity

## Abstract

Although prokaryotic DNA methylation investigations have long focused on immunity against exogenous DNA, it has been recently recognized that DNA methylation impacts gene expression and phase variation in Streptococcus pneumoniae and Streptococcus suis. A comprehensive analysis of DNA methylation is lacking for beta-hemolytic streptococci, and thus we sought to examine DNA methylation in the major human pathogen group A Streptococcus (GAS). Using a database of 224 GAS genomes encompassing 80 *emm* types, we found that nearly all GAS strains encode a type I restriction modification (RM) system that lacks the *hsdS′* alleles responsible for impacting gene expression in S. pneumoniae and S. suis. The GAS type I system is located on the core chromosome, while sporadically present type II orphan methyltransferases were identified on prophages. By combining single-molecule real-time (SMRT) analyses of 10 distinct *emm* types along with phylogenomics of 224 strains, we were able to assign 13 methylation patterns to the GAS population. Inactivation of the type I RM system, occurring either naturally through phage insertion or through laboratory-induced gene deletion, abrogated DNA methylation detectable via either SMRT or MinION sequencing. Contrary to a previous report, inactivation of the type I system did not impact transcript levels of the gene (*mga*) encoding the key multigene activator protein (Mga) or Mga-regulated genes. Inactivation of the type I system significantly increased plasmid transformation rates. These data delineate the breadth of the core chromosomal type I RM system in the GAS population and clarify its role in immunity rather than impacting Mga regulon expression.

**IMPORTANCE** The advent of whole-genome approaches capable of detecting DNA methylation has markedly expanded appreciation of the diverse roles of epigenetic modification in prokaryotic physiology. For example, recent studies have suggested that DNA methylation impacts gene expression in some streptococci. The data described herein are from the first systematic analysis of DNA methylation in a beta-hemolytic streptococcus and one of the few analyses to comprehensively characterize DNA methylation across hundreds of strains of the same bacterial species. We clarify that DNA methylation in group A Streptococcus (GAS) is primarily due to a type I restriction modification (RM) system present in the core genome and does not impact *mga*-regulated virulence gene expression, but does impact immunity against exogenous DNA. The identification of the DNA motifs recognized by each type I RM system may assist with optimizing methods for GAS genetic manipulation and help us understand how bacterial pathogens acquire exogenous DNA elements.

## INTRODUCTION

Methylation of DNA impacts a broad variety of physiological functions in both prokaryotic and eukaryotic organisms. In prokaryotes, DNA methylation has mainly been investigated for its role in innate immunity although there is increasing recognition that DNA methylation can also modulate gene expression. Eukaryotes typically methylate cytosines on C5 (m5C). While prokaryotes also contain m5C, N6-methyladenine (m6A) is the predominant form of prokaryotic methylation, and N4-methylcytosine (m4C) is found exclusively in bacterial genomes (1, 2). In prokaryotes, base methylation is catalyzed by methyltransferases (MTases) that are typically components of restriction modification (RM) systems (3, 4). RM systems are widespread, being found in >90% of all sequenced prokaryotes (5, 6).

Bacterial RM systems are classified into four types based on components, sequence specificity, cofactors, and cleavage position (7, 8). The type I RM systems are hetero-oligomeric and contain an MTase (HsdM), a restriction endonuclease (REase [HsdR]), and a DNA specificity subunit (HsdS). HsdS combines with HsdM and HsdR to methylate and cleave unmethylated DNA targets, respectively. The DNA motifs targeted by type I RM systems are bipartite, and cleavage occurs at large distances from their binding sites (9). Type II RM systems bind short motifs, cleave within or close to the binding site, and are the most-studied systems, commercialized and used for genetic manipulation of DNA in laboratory protocols (10). While some type II systems consist of a single protein that functions as an MTase and REase (type IIG, IIB, and IIC), most have separate MTases and REases (11). Additionally, a large number of type II systems consist of only an orphan MTase (11, 12). Of these, the Dam and CcrM methylases are well-studied examples (13, 14). In type III systems, *res* and *mod* encode the REase and MTase, respectively. These systems recognize short palindromic motifs and cleave outside the binding site (15). Some type I and type III RM systems are phase variable and can alter their methylation patterns reversibly by swapping specificity components (16). In contrast to the groups described above, type IV systems have only an REase. DNA targets containing methylated, hydroxymethylated, or glucosyl-hydroxymethylated bases are cleaved (7).

In addition to distinguishing between self and nonself, prokaryotic methylation affects a variety of cellular functions, such as DNA mismatch repair, cell cycle control, transcriptional regulation, and virulence (13, 17). Phase-variable RM systems impact the virulence of numerous clinically important organisms, including Helicobacter pylori (18), Neisseria meningitidis (19), Haemophilus influenzae (20), and Streptococcus pneumoniae (21, 22). Phase variation in Streptococcus pneumoniae and Streptococcus suis is achieved by recombination between tandemly arranged HsdS subunits in the type I RM system (21, 23). In contrast, there has been less study of DNA methylation in beta-hemolytic streptococci. A phage-borne type II m5C MTase was the first characterized MTase in Streptococcus pyogenes, also known as group A Streptococcus (GAS). It was found to protect genomic DNA from digestion by SmaI and was speculated to function in maintaining erythromycin-resistant GAS populations (24). Nye et al. (25) recently reported that a type I RM system in *emm28* GAS was responsible for the majority of DNA methylation and that inactivation of the system decreased transcription of the gene (*mga*) encoding the key multigene activator (Mga) transcriptional regulator. Conversely, inactivation of the type I RM system in an *emm1* GAS strain did not impact virulence gene transcript levels (26). Currently, a systematic examination of RM systems in GAS is not available.

The large number of available GAS genome sequences and the clustering of GAS strains by *emm* types potentially make GAS a useful organism for understanding the distribution and function of RM systems among beta-hemolytic streptococci. Herein, we took a combined bioinformatic and biological approach to study GAS RM systems. We found that the core GAS genome contains a single type I RM system and that variable type II RM systems are located on prophages. Through PacBio single-molecule real-time (SMRT) sequencing, we compiled the various DNA motifs methylated by the type I RM systems in different GAS strains and found that GAS *emm* types that cluster based on their core gene alignment do not necessarily harbor similar methylation target specificities. Finally, we inactivated the type I MTase in two distinct *emm* types and found that the type I MTases did not contribute to *mga* or Mga-regulated virulence gene transcript levels but did impact efficiency of DNA uptake. Taken together, these data are the first comprehensive analysis of RM systems in GAS and suggest that the type I RM systems ubiquitously present in GAS are likely primarily involved in innate immunity rather than modulation of genes that are Mga regulated.

## RESULTS

### Overview of RM systems in GAS and closely related streptococci.

**FIG 1:**
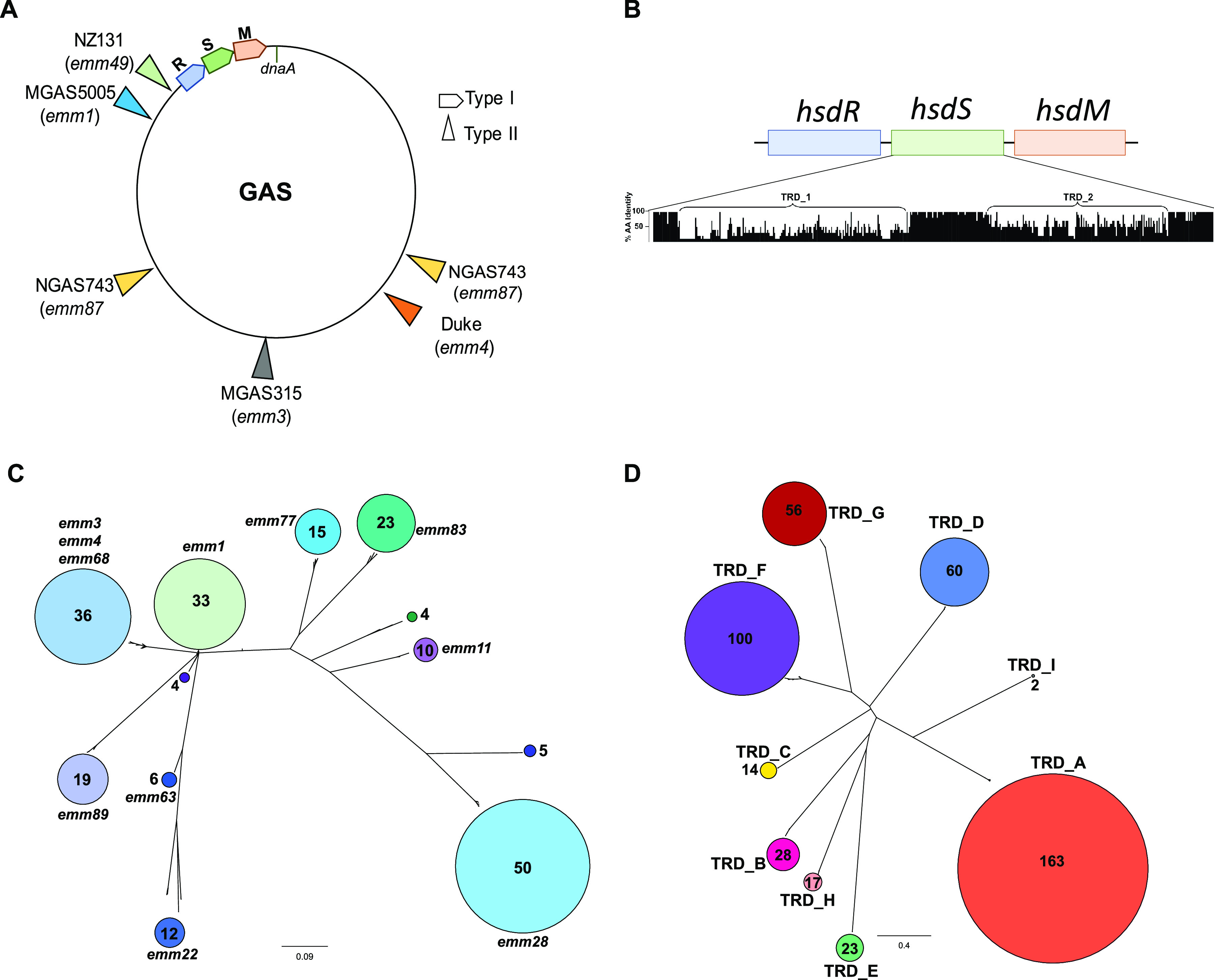
Overview of GAS type I RM systems. (A) A schematic figure shows the location of the type I RM system on the GAS chromosome. Locations of selected type II RM systems relative to *dnaA* are also shown, and the respective GAS strain and *emm* type are indicated in parentheses. (B) Alignment of HsdS sequences from the 10 most common target recognition domain (TRD) patterns identified in our GAS population. The percentage of amino acid identity across each position was calculated using Jalview (72) and is displayed (0 to 100%) on the *y* axis of the histogram. The *x* axis indicates amino acid positions. (C and D) Unrooted, neighborhood-joining trees based on (C) full-length HsdS sequences and (D) TRD sequence alignment from the 224 GAS strains in this study. GAS *emm* types dominating each HsdS cluster are indicated. The number of strains (C) and TRDs (D) in each cluster is indicated within the colored circles and reflected by circle size.

Only a handful of studies of RM systems using a large number of strains from the same bacterial species have been reported, and none in beta-hemolytic streptococci (27, 28). We analyzed 224 GAS genomes from 80 different *emm* types to understand the diversity of RM systems (see Table S1 in the supplemental material). We found that GAS strains generally contain a single, core chromosomal type I RM system, along with variable type II systems located in prophages (Fig. 1A). We found no evidence of type IIG, type III, or type IV systems. The type I RM system was consistently present at the same location in the GAS genome (Fig. 1A) and exhibited an *hsdRSM* arrangement. There were isolated GAS strains in which the type I system was either interrupted by a prophage or completely deleted, as has been previously reported (29, 30).

10.1128/mSphere.00799-21.7 TABLE S1 List of GAS strains used for TRD and phylogenomic analysis. Download Table S1, XLSX file, 0.03 MB. Copyright © 2021 DebRoy et al. 2021 DebRoy et al. https://creativecommons.org/licenses/by/4.0/ This content is distributed under the terms of the Creative Commons Attribution 4.0 International license.

HsdR and HsdM proteins were highly conserved among GAS strains (>95% identity at the amino acid level for both proteins across the 224 genomes), while GAS HsdS was highly variable, as will be discussed later. GAS HsdR is nearly identical (93% identical and 96% similar at the amino acid level) to HsdR present in 10 Streptococcus agalactiae (also known as group B Streptococcus [GBS]) strains, although the vast majority of sequenced GBS strains did not contain a GAS HsdR homolog (Table 1). GAS HsdR was ∼70% similar to HsdR homologs present in various viridans group streptococci (VGS) (i.e., S. pneumoniae) and 64/78% identical/similar to a single strain of Streptococcus dysgalactiae (Streptococcus sp. strain 714). Analogous to HsdR, GAS HsdM was 97/99% identical/similar to HsdM homologs present in ∼30 GBS strains, although like HsdR, most GBS strains did not contain a GAS HsdM homolog (Table 1). Many GBS strains that contained a GAS HsdM homolog lacked an HsdR homolog and evidenced loss of genetic material at the location of the type I RM system site (see Fig. S1 in the supplemental material). GAS HsdM had ∼74/86% identity/similarity to three *S. dygalactiae* strains as well as many other VGS (Table 1). Taken together, these data showed that the GAS HsdR and HsdM proteins are not widely shared among closely related beta-hemolytic streptococci such as Streptococcus equi or S. dysgalactiae subsp. *equisimilis* but do occasionally have close homologs in sporadic GBS isolates.

**TABLE 1:** Comparison of HsdR and HsdM between GAS and other streptococci

S. pyogenes/GAS	% of identity or similarity to GAS HsdR or HsdM^*a*^					
	S. agalactiae/GBS^*b*^		VGS		S. dysgalactiae ^*b*^	
	Identity	Similarity	Identity	Similarity	Identity	Similarity
HsdR	93	96	70	70	64	78
HsdM	97	99	74	86	47	86

a The percentages of identity and similarity indicated are at the amino acid level. VGS, viridans group streptococci.

b Homologs are present in only a few strains.

10.1128/mSphere.00799-21.1 FIG S1 Schematic representation comparing the architecture of the GAS type I RM system to that of Streptococcus agalactiae (also known as group B Streptococcus). Download FIG S1, EPS file, 1.2 MB. Copyright © 2021 DebRoy et al. 2021 DebRoy et al. https://creativecommons.org/licenses/by/4.0/ This content is distributed under the terms of the Creative Commons Attribution 4.0 International license.

### Analysis of the GAS HsdS protein and its tandem recognition domains.

The GAS type I RM system contains a single *hsdS* gene. In contrast, the type I RM system from many S. pneumoniae and some S. suis strains previously shown to affect gene expression contains two pseudogenes, *hsdS′* and *hsdS′′*, which provide a scaffold for recombination and thus alteration of HdsS targeting (see Fig. S2 in the supplemental material) (21, 23). Alignment of the HsdS protein from 224 GAS genomes revealed several main findings. First, there was significantly less HsdS protein homology shared across the GAS genomes relative to HsdR and HsdM, with HsdS from different strains having as little as 37% identity over the full length of the protein (Fig. 1B). Second, comparison of the HsdS protein revealed clear clustering, with particular clusters often harboring strains of more than one *emm* type (Fig. 1C). Finally, the diversity of the HsdS protein was located in two distinct regions, which we will refer to as the TRD1 and TRD2 positions, consistent with these areas being target recognition domains (TRDs), which function to detect a specific combination of a bipartite DNA target sequence (Fig. 1B) (9). The presumed TRDs are flanked by and separated by relatively conserved regions with the length of the inter-TRD conserved region dictating the distance between the two halves of the target sequence (31).

10.1128/mSphere.00799-21.2 FIG S2 Comparison of the type I RM systems of GAS, Streptococcus pneumoniae, and Streptococcus suis. Two examples of S. pneumoniae are included to display different arrangements of the *hsdS′* and *hsdS′′* genes as observed in sequenced strains. Download FIG S2, EPS file, 1.2 MB. Copyright © 2021 DebRoy et al. 2021 DebRoy et al. https://creativecommons.org/licenses/by/4.0/ This content is distributed under the terms of the Creative Commons Attribution 4.0 International license.

**FIG 2:**
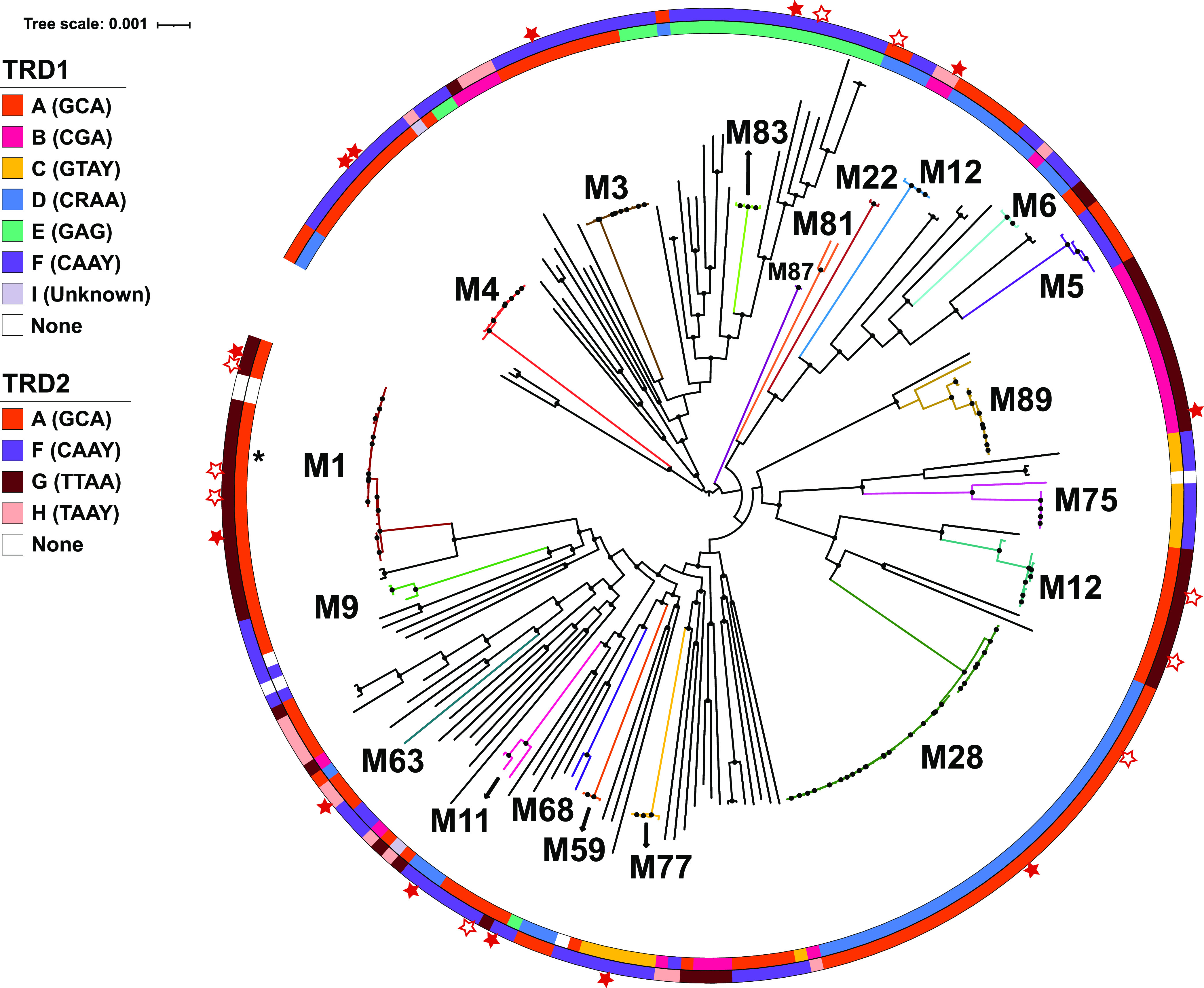
Correlation between GAS TRD alleles and phylogeny inferred from core gene alignment. The maximum likelihood phylogenetic tree was created from a core gene alignment of the 224 GAS genomes. Inner and outer circles are color coordinated to indicate the TRD present in positions 1 and 2 of HsdS, respectively. The half-site of the target recognized by each TRD is listed in parentheses within the legend. Closed stars indicate strains that were sequenced by PacBio in this study. Open stars are GAS strains for which PacBio data are available on REBASE (73). Major GAS *emm* types are indicated, and isolates of the same *emm* type tend to cluster, with the exception of *emm12*. Black dots on internal nodes indicate >95% bootstrap support. The single black star along the inner circle denotes a GAS strain that clusters with the *emm1* strains but belongs to *emm204*.

Given that the presumed TRDs account for HsdS diversity, we next focused on analyzing these individually. Based on TRD sequence alignment and subsequent clustering analysis, we identified nine distinct TRDs that varied widely in their prevalence (Fig. 1D). Within clusters, the TRD amino acid compositions were highly similar—generally 99 to 100% identity but always >92%. Conversely, the amino acid identity levels were typically 15% or less between TRDs from distinct clusters. Of the 9 unique TRDs, 7 occurred in either the N-terminus (i.e., TRD1) or C-terminus (i.e., TRD2) position. TRD_B, -C, -D, -E, and -I occurred only at position 1, and the TRD_G and -H alleles were only found at position 2. TRD_A and TRD_F exhibited domain movement, as previously described (32). Specifically, TRD_A, which was typically found in the TRD1 position, was located in the TRD2 position for several strains from different GAS *emm* types (Fig. 2; see Fig. S3 in the supplemental material). Conversely, TRD_F, which usually occurred at a TRD2 position, was present in the TRD1 position for all five *emm5* strains (Fig. 2). Interestingly, TRD_F was the only TRD allele that was found to occur in positions 1 and 2 of the same HsdS protein (TRD1/2 [F/F]) in four different GAS *emm* types (Fig. 2).

10.1128/mSphere.00799-21.3 FIG S3 A schematic representation of the movement of TRD domains within GAS strains (*emm1*/*emm28*) and between GAS and GBS strains compared using EasyFig (67). Download FIG S3, EPS file, 0.7 MB. Copyright © 2021 DebRoy et al. 2021 DebRoy et al. https://creativecommons.org/licenses/by/4.0/ This content is distributed under the terms of the Creative Commons Attribution 4.0 International license.

There were 13 distinct TRD combinations among the analyzed GAS strains, with occasional strains lacking one or more TRDs or even the entire HsdRSM system (Fig. 2; Table S1). The majority of strains of the same *emm* type contained the same TRD1/2 combination, with the main exception being *emm12* strains, which had two completely different TRD1/2 combinations (AG and DA). Similar variation of TRD1/2 combination between strains of the same *emm* type was also observed for *emm22*, *emm25*, *emm44*, *emm64*, *emm68*, *emm70*, *emm75*, *emm77*, *emm78*, and *emm92* isolates. When analyzed by *emm* type, the EF combination was the most common, being present in 17 *emm* types, with AF (16 *emm* types), AG (13 *emm* types), and DA (10 *emm* types) being observed in at least 10 *emm* types. Conversely, the FA and FH combinations were only observed in a single *emm* type.

To determine whether the TRD combinations correlated with core genome phylogenies, we created a maximum likelihood phylogenetic tree inferred from core gene alignment and then layered the TRD combinations on top of this tree (Fig. 2). Interestingly, GAS strains that are quite distinct at the whole-genome level (e.g., *emm1* and *emm12*) share identical TRD1/2 alleles. Conversely, *emm* types that are closely related based on core gene phylogeny (e.g., *emm5* and *emm6*) can have completely different TRD1/2 alleles, consistent with the occurrence of horizontal gene transfer (HGT) among different GAS *emm* types. The two groups of aforementioned *emm12* strains with distinct TRD combinations also were significantly different at the core gene level, suggesting that horizontal transfer of the *emm* gene may have occurred (33).

Accessory genome elements account for ∼10% of the average GAS genome and encode a variety of critical GAS virulence factors, such as pili, cell surface molecules, and superantigens (34). The exogenous nature of many of these non-core chromosomal elements suggests that their presence could be influenced by the activity of the type I RM system. Therefore, we next asked whether there was a relationship between TRD composition and accessory gene content. As shown in Fig. S4 in the supplemental material, we did observe some clustering of accessory genes by TRD group, which was primarily driven by strains of identical *emm* types. When strains of distinct *emm* types were considered, we did not discern clustering of accessory gene content relative to TRD composition (e.g., yellow dots representing the AF TRD combination are present both in the middle and lower left quadrants of Fig. S4). We conclude that TRD composition alone is not determinative of accessory gene content.

10.1128/mSphere.00799-21.4 FIG S4 GAS TRD alleles do not correlate with accessory genome composition. A t-SNE plot indicates the distribution of TRD1/2 combinations in the accessory genomes of 224 GAS strains. Each dot indicates a single isolate’s accessory genome (gene content shared in <99% genomes), with color indicating TRD pattern assignment per the legend. *emm* types that clustered by accessory genome content are labeled on the figure. An asterisk indicates one isolate belonging to *emm204* that clusters with *emm1* isolates. Download FIG S4, EPS file, 0.5 MB. Copyright © 2021 DebRoy et al. 2021 DebRoy et al. https://creativecommons.org/licenses/by/4.0/ This content is distributed under the terms of the Creative Commons Attribution 4.0 International license.

### GAS TRD composition and recombination.

Recombination events within GAS strains as well as between GAS and other hemolytic streptococci have been reported (35, 36). Given that type I RM systems are thought to limit recombination (28), we next sought to determine whether the compositions of the GAS TRDs were identical between *emm* types previously identified as having undergone recombination. The best-described example of recombination to date among GAS is between *emm1* and *emm12* strains and involves a 36-kb segment of DNA, including the *nga*-*slo* region (35). As noted above, we identified two distinct *emm12* TRD combinations. Examination of the *emm12* genomes showed significant variation in the *nga*-*slo* locus between the two clusters. The *emm12* strains that contain an *nga*-*slo* region nearly identical to *emm1* strains also have the same TRD combination (AG) as *emm1* strains. Another major identified recombination occurred between GBS and *emm28* strains (36). Consistent with TRD composition being important for recombination events, *emm28* strains contain TRD_D in the HsdS TRD1 position, which is the TRD allele present with high homology in a limited number of GBS isolates. The TRD2 *emm28* allele TRD_A was not identified in any GBS isolates (Fig. S3). No highly similar TRDs were identified in strains of streptococci closely related at the whole-genome level to GAS such as S. dysgalactiae.

### Use of SMRT sequencing to identify HsdS TRD allele methylation specificity.

Given that there were significant clusters of GAS HsdS proteins, we next sought to determine the target recognition sites (TRSs) for methylation by the various HsdS isoforms. We selected for genome-wide methylation analysis 10 different GAS strains that included representatives of each cluster that had more than five isolates in our HsdS analysis (Fig. 1C). All reads from the SMRT sequencing were aligned to the respective reference genomes (see Table S2a in the supplemental material) to identify the location and type of methylation. Methylation was detected in all GAS genomes sequenced, and the most prevalent type of methylation observed was m6A (see Table 3 below). For several of the sequenced strains, we identified additional methylation events (Table S2b) with low modification quality values (modQV) compared to that observed for m6A. A previous study reported similar observations and suggested that these events likely reflect “noise” in the SMRT data set (25).

10.1128/mSphere.00799-21.8 TABLE S2 (a) List of strains analyzed by PacBio and reference genomes used. (b) List of DNA motifs with non-m6A base modification detected by PacBio. Download Table S2, XLSX file, 0.01 MB. Copyright © 2021 DebRoy et al. 2021 DebRoy et al. https://creativecommons.org/licenses/by/4.0/ This content is distributed under the terms of the Creative Commons Attribution 4.0 International license.

We then analyzed sequence motifs that recurred at these m6A sites to derive the target recognition site (TRS) for the MTase in each genome. We identified 9 different TRSs with more than 97% of the identified motifs being methylated in each sequenced genome (Table 3). Given the bipartite nature of the TRS and the correspondence of each half of the motif to TRD1 and -2 in HsdS, we were able to assign the half-motif recognized by all the TRDs identified from our HsdS analysis, with the exception of TRD_I. The distributions of TRDs and TRSs are shown in Fig. 2.

### The type I RM system is the major source of methylation.

**FIG 3:**
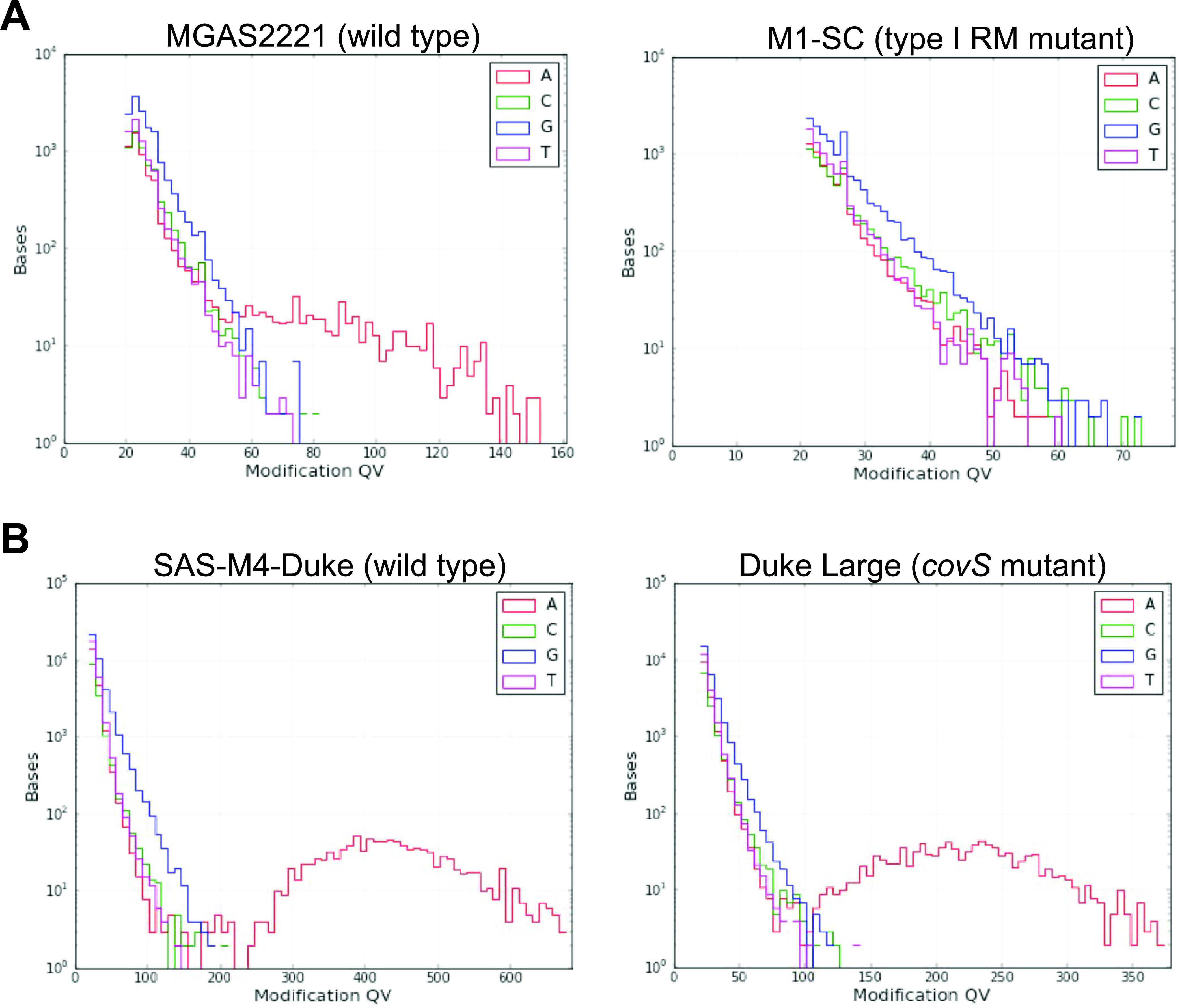
Methylation events detected in various GAS strains. Comparison of modification quality value (modQV) histograms indicating methylation events detected by PacBio sequencing between (A) *emm1* strains MGAS2221 and M1-SC and (B) *emm4* strains SAS-M4-Duke and Duke Large. modQV values are indicated on the *x* axis, and the numbers of bases are displayed on the *y* axis. The lines are color-coded for each nucleotide.

**FIG 4:**
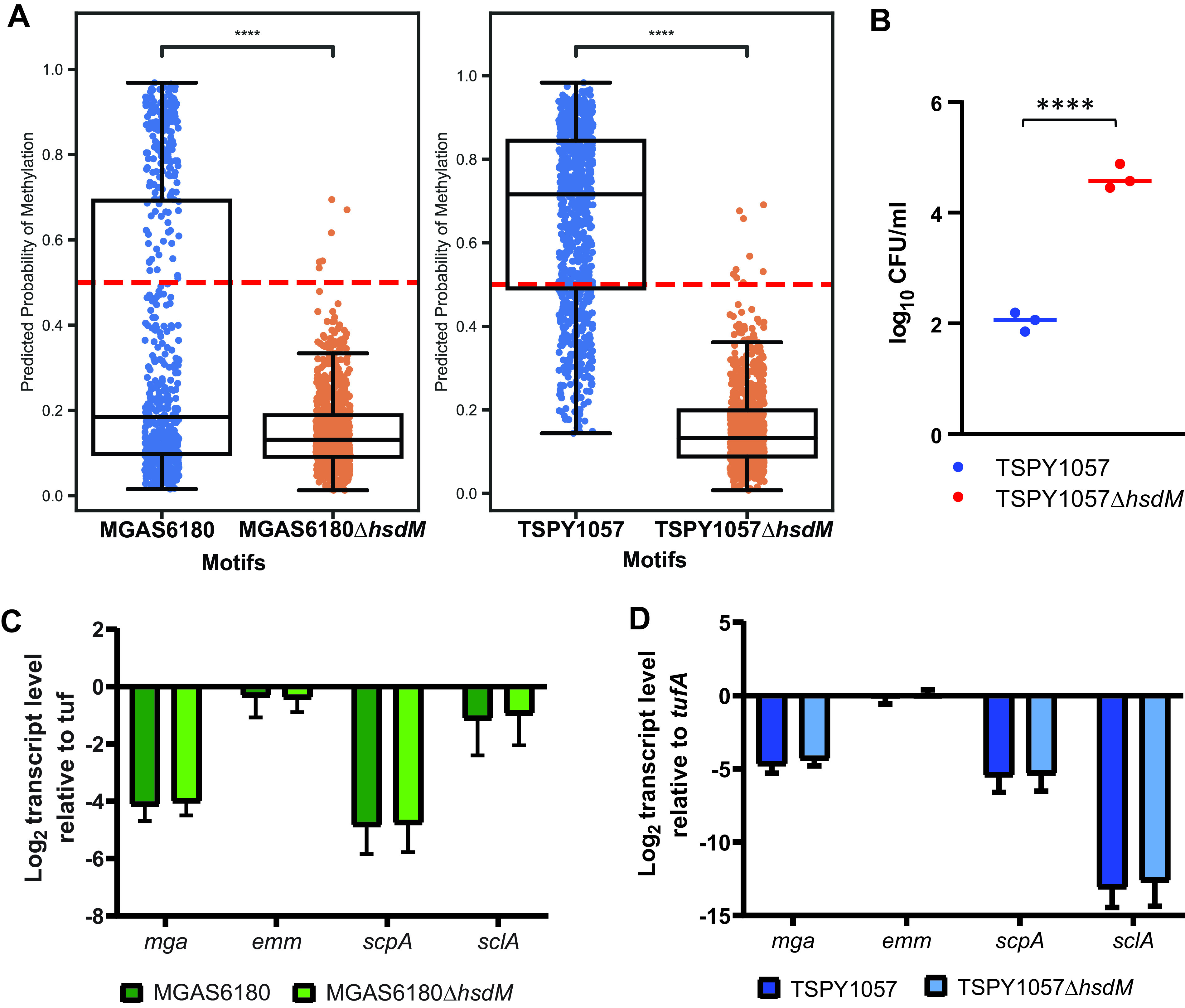
Characterization of impact of *hsdM* inactivation on GAS methylation, transformation, and gene expression. (A) Comparison of methylation events detected by the Caller neural network model using ONT long-read sequencing data between wild-type (blue dots) and Δ*hsdM* mutant (orange dots) strains of MGAS6180 (*emm28*) and TSPY1057 (*emm87*), respectively. The dotted red line in each subpanel indicates a probability of 0.5. Welch’s *t* test of independent samples with a multiple-sample Bonferroni correction was performed for each wild-type strain and respective Δ*hsdM* group, with **** indicating statistically significant difference between the strains at *P* ≤ 0.0001. (B) Comparison of transformation efficiency between TSPY1057 and its Δ*hsdM* mutant. GAS cells were transformed on three separate occasions, and the numbers of colonies detected are shown as log_10_ CFU/ml per μg DNA. ****, statistically significant difference between the strains at *P* ≤ 0.0001 by unpaired t test (C and D) TaqMan qRT-PCR analysis of the impact of *hsdM* deletion on transcript levels of the gene encoding the multigene activator (*mga*) and Mga-regulated genes in (C) MGAS6180 and (D) TSPY1057. TaqMan qRT-PCR data are means ± standard deviations from two biological replicates, with two technical replicates, done on 2 separate days.

Given that GAS strains consistently harbor only the single type I RM system, we sought to determine if all of the observed methylation was due to these MTases. To this end, we performed SMRT sequencing of an *emm1* GAS strain with a prophage insertion in *hsdRSM* (37). We found that this strain, M1-SC, completely lacked m6A, consistent with the idea that the type I system is the major methylation system in GAS (Table 3; Fig. 3A). To investigate further, we generated targeted knockouts of *hsdM* in *emm28* (MGAS6180) and *emm87* (TSPY1057) strains (Table 2) and confirmed the absence of spurious mutations by whole-genome sequencing. Deletion of the *hsdM* gene did not impact the growth of these strains in standard laboratory media (see Fig. S5A in the supplemental material). We also confirmed the absence of *hsdM* transcripts in the mutant strains by TaqMan quantitative real-time PCR (qRT-PCR) (Fig. S5B). We used Oxford Nanopore Technologies (ONT) sequencing and a neural network classifier (38) to detect m6A methylation and determine if *hsdM* knockouts would reduce methylation detected in GAS genomes. There was m6A methylation in both wild-type strains MGAS6180 and TSPY1057, while deletion of *hsdM* resulted in statistically significant reduction in methylation for both (Fig. 4A). We next assessed the ability of the *emm87* strain TSPY1057 and its *hsdM* mutant to incorporate DNA by electroporation. We found that the TSPY1057 Δ*hsdM* mutant yielded ∼500-fold more transformants when electroporated with the pLZ12 plasmid (39) compared to the wild-type strain (Fig. 4B). Similar increases in transformation efficiency of strains lacking the type I RM system have been reported for *emm1* and *emm28* GAS (25, 26, 30). Taken together, we conclude that the type I RM system is responsible for methylation observed in GAS strains, and disruption of this system improves entry of exogenous DNA under laboratory conditions.

**TABLE 2:** Bacterial strains used in this study

Strain	Description	Reference
MGAS2221	Invasive clinical isolate, *emm1*	41
M1-SC-1	Clinical isolate, *emm1*	37
MSPY1	Clinical isolate, *emm89*	69
SAS-M4-Duke	Clinical isolate, *emm4*	43
RLGH	Clinical isolate, *emm4*	43
Duke Large	Invasive clinical isolate, coisolated with SAS-M4-Duke, inactive CovS	44
Duke Δ*covS*	Isogenic mutant of SAS-M4-Duke, CovS inactive	44
MGAS10870	Clinical isolate, *emm3*	70
TSPY416	*emm68*	This study
TSPY125	Clinical isolate, *emm83*	This study
TSPY155	Clinical isolate, *emm11*	62
TSPY453	Clinical isolate, *emm77*	62
TSPY1309	Clinical isolate, *emm63*	This study
TSPY136	Clinical isolate, *emm22*	This study
MGAS6180	Clinical isolate, *emm28*	36
MGAS6180 Δ*hsdM*	Isogenic *hsdM* mutant	This study
TSPY1057	Clinical isolate, *emm87*	71
TSPY1057 Δ*hsdM*	Isogenic *hsdM* mutant	This study

10.1128/mSphere.00799-21.5 FIG S5 Isogenic *hsdM* mutants of *emm28* and *emm87* GAS. (A) Growth of MGAS6180 and TSPY1057 and their isogenic *hsdM* mutants in THY broth. (B) TaqMan qRT-PCR confirms lack of *hsdM* transcripts in isogenic Δ*hsdM* strains. Data show the mean ± standard deviation from two biological replicates, with two technical replicates, done on separate days. Download FIG S5, EPS file, 0.9 MB. Copyright © 2021 DebRoy et al. 2021 DebRoy et al. https://creativecommons.org/licenses/by/4.0/ This content is distributed under the terms of the Creative Commons Attribution 4.0 International license.

### Inactivation of the type I RM system does not alter transcript levels of *mga* or Mga-regulated virulence genes.

A previous study of MEW123 (*emm28*) demonstrated reduced transcript levels of *mga* and other genes in the Mga regulon upon inactivation of the type I RM system (25). However, a recent report found no differences in the transcript levels of known GAS virulence factor-encoding genes when comparing an isogenic *emm1 hsdM* mutant to its wild-type parent (26). To address a potential role of methylation in expression of *mga* and its regulon and the possibility of *emm*-specific differences, we performed targeted gene transcript-level analysis of both MGAS6180 (*emm28*) and TSPY1057 (*emm87*) compared to their respective isogenic Δ*hsdM* mutants. In accordance with the *emm1* study and unlike the *emm28* report, we found no difference in the transcript levels of *mga* or Mga-regulated genes upon inactivation of the type I RM system (Fig. 4C and D).

### Analysis of intra-*emm* type methylation patterns.

It is well known that GAS strains belonging to the same *emm* type (and likely with identical TRD1/2 alleles) can exhibit differing virulence attributes, either inherently or due to naturally occurring alterations (40). Given the known relationship between epigenetics and virulence in other streptococci (21, 22), it is possible that epigenetics might play a role in GAS virulence. To address this question, we performed SMRT sequencing to compare GAS strains of the same *emm* type and TRD1/2 combination, but with distinct virulence phenotypes. First, we compared the methylation patterns of the *emm1* strains SF370 and MGAS2221, which represent two distinct *emm1* clades that are well known to differ markedly in their virulence (40). Another established difference between these two strains is that MGAS2221 readily develops hypervirulent mutants that harbor changes in the control of virulence (CovRS) two-component gene regulatory system, while the same is not observed in SF370 (41, 42). Consistent with the TRDs being the determinative factor of GAS methylation, we found that the methylation patterns of SF370 and MGAS2221 were nearly identical (Table 3; see Fig. S6A in the supplemental material). We also compared the *emm4* strains SAS-M4-Duke and RLGH, which like the *emm1* strains differ in the spontaneous occurrence of hypervirulent CovRS mutants, and again found identical methylation patterns (Fig. S6B). To address whether a CovS mutant has altered methylation, we studied three *emm4* strains, SAS-M4-Duke (wild type), a spontaneous CovS mutant derived from Duke (Duke Large), and an isoallelic CovS mutant of Duke (Duke Δ*covS*) (43, 44). SMRT analysis of the three strains showed no difference in the extent of methylation or in the motifs targeted, suggesting that genome-wide methylation is not altered upon inactivation of the CovRS system (Table 3; Fig. 3B). Taken together, we conclude that methylation variation does not account for differences in virulence attributes observed among the tested GAS strains with identical TRD alleles.

**TABLE 3:** Methylation identified in the genomes of GAS strains from different *emm* types

GAS strain	*emm* type	Motif string	Position modified	Type	% of motifs detected	No. of motifs detected	No. of motifs in genome	Mean modQV
MGAS2221	1	GCANNNNNNTTAA	3	m6A	98.0	337	344	79.09
		TTAANNNNNNTGC	3	m6A	97.7	336	344	77.17

SF370	1	TTAANNNNNNTGC	3	m6A	98.8	331	335	75.26
		GCANNNNNNTTAA	3	m6A	97.9	328	335	76.43

M1-SC	1	ND						

MGAS10870	3	CAAYNNNNNNTGC	3	m6A	100.0	524	524	513.45
		GCANNNNNNRTTG	3	m6A	99.8	523	524	493.54

SAS-M4-Duke	4	CAAYNNNNNNTGC	3	m6A	100.0	542	542	143.86
		GCANNNNNNRTTG	3	m6A	100.0	542	542	142.56

RLGH	4	CAAYNNNNNNTGC	3	m6A	100.0	542	542	307.29
		GCANNNNNNRTTG	3	m6A	99.8	541	542	299.71

Duke Large	4	CAAYNNNNNNTGC	3	m6A	100.0	542	542	228.72
		GCANNNNNNRTTG	3	m6A	100.0	542	542	225.93

Duke Δ*covS*	4	GCANNNNNNRTTG	3	m6A	98.2	532	542	68.37
		CAAYNNNNNNTGC	3	m6A	96.5	523	542	68.08

TSPY155	11	CRAANNNNNNRTTG	4	m6A	99.8	462	463	283.43
		CAAYNNNNNNTTYG	3	m6A	99.6	461	463	312.70

TSPY136	22	TAAYNNNNNNTCG	3	m6A	100.0	241	241	285.17
		CGANNNNNNRTTA	3	m6A	100.0	241	241	258.73

MGAS6180	28	CRAANNNNNNNTGC	4	m6A	99.5	414	416	203.92
		GCANNNNNNNTTYG	3	m6A	99.0	412	416	229.83

TSPY1309	63	TAAYNNNNNNTGC	3	m6A	100.0	537	537	525.49
		GCANNNNNNRTTA	3	m6A	99.8	536	537	474.90

TSPY416	68	CAAYNNNNNNTGC	3	m6A	100.0	513	513	270.36
		GCANNNNNNRTTG	3	m6A	99.8	512	513	259.95

TSPY453	77	GTAYNNNNNRTTG	3	m6A	100.0	190	190	412.97
		CAAYNNNNNRTAC	3	m6A	100.0	190	190	404.43

TSPY125	83	CAAYNNNNNNCTC	3	m6A	98.9	345	349	476.50
		GAGNNNNNNRTTG	2	m6A	98.0	342	349	458.08

MSPY1	89	TTAANNNNNNTCG	3	m6A	100.0	179	179	312.57
		CGANNNNNNTTAA	3	m6A	99.4	178	179	280.25

10.1128/mSphere.00799-21.6 FIG S6 Modification quality value (QV) histograms indicating methylation events detected by PacBio sequencing between (A) *emm1* strains MGAS2221 and SF370 and (B) *emm4* strains SAS-M4-Duke and RLGH. Modification quality values (modQV) are indicated on the *x* axis, and the number of bases is displayed on the *y* axis. The lines are color-coded for each nucleotide. Download FIG S6, TIF file, 2.5 MB. Copyright © 2021 DebRoy et al. 2021 DebRoy et al. https://creativecommons.org/licenses/by/4.0/ This content is distributed under the terms of the Creative Commons Attribution 4.0 International license.

### GAS type II RM systems.

**FIG 5:**
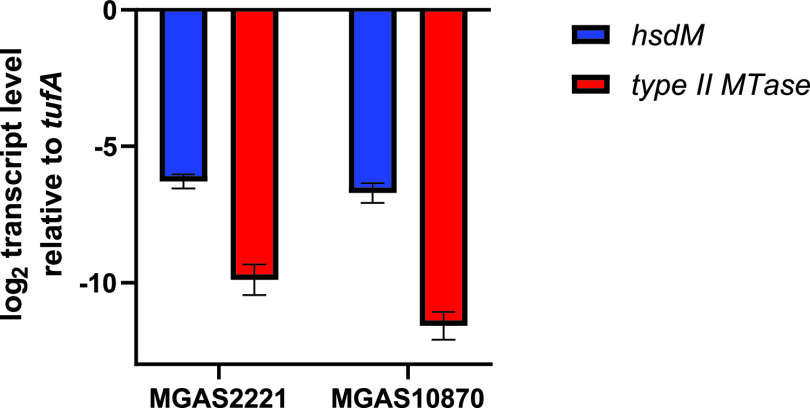
Genes encoding the type I MTase have higher transcript levels than those encoding type II RM MTases. Shown are the results from analysis of transcript levels of genes encoding the MTase of the type I and type II RM systems in representative *emm1* (MGAS2221) and *emm3* (MGAS10870) strains. The data shown are means ± standard deviations from two biological replicates, with two technical replicates, done on 2 separate days.

Type II RM systems typically consist of an MTase and an REase, encoded by a single or separate genes (11). However, it is well recognized that a large number of type II orphan methyltransferases can be present on bacteriophages and other mobile genetic elements (11, 12, 45). We identified only a single type II system in our 224 GAS strains that contained both REase and MTase enzymes. This system was present on a prophage in the *emm6* strain MGAS10394, the *emm1* strain 10-85, and the *emm75* strain TSPY208 and has been previously characterized as important for cytosine methylation (m5C) in strain MGAS10394 (24). The vast majority of sequenced GAS strains contained at least one and up to three orphan type II methyltransferases present on endogenous prophages. The presence of these type II MTase-containing prophages varies both among and within *emm* types (Fig. 1A), and these prophages also typically contain virulence genes encoding superantigens (e.g., SpeC or Ssa) or DNases (e.g., SdaI or SpdI) (46, 47). In one strain (MGAS10750 [*emm4*]), a prophage encoding a type II orphan methyltransferase had integrated into and inactivated the type I RM system (29). Despite the widespread presence of the type II orphan MTases, we did not observe any m5C methylation in our SMRT analyses. It is well known that m5C is not as readily detected by SMRT sequencing as m6A, and thus increasing sequencing depth is needed for confident detection (48). We achieved sequencing depths of >250 recommended for m5C and still did not identify any m5C modification (49). We hypothesized that the lack of m5C might be due to very low or absent expression of the type II MTases under the conditions in which samples were collected for SMRT sequencing. Consistent with this hypothesis, we found little to no transcripts of the genes encoding type II MTase genes of MGAS2221 (*emm1*) and MGAS1870 (*emm3*), respectively (Fig. 5). Given that genes carried on prophages can be induced (50, 51), it remains formally possible that the type II orphan MTases do contribute to the GAS methylome under different conditions.

## DISCUSSION

The advent of whole-genome sequencing (WGS) approaches capable of detecting DNA methylation has facilitated high-throughput analyses of epigenetic modifications, which in turn has greatly facilitated understanding of how DNA methylation impacts different aspects of prokaryotic physiology. Herein, we used a combination of WGS and large-scale phylogenomics to systematically characterize the restriction modification (RM) systems of 224 strains of the major human pathogen group A Streptococcus (GAS). We found that a single type I RM system present in the GAS core genome is responsible for methylation detectable by different WGS approaches and is distinct from the type I systems in S. pneumoniae and S. suis recently shown to impact global gene expression. Our analysis of the type I system defined the DNA methylation motifs of 13 combinations of nine TRDs present and correlated GAS TRD composition with the GAS pangenome.

Our conclusion that the type I RM system present on the GAS chromosome is responsible for most, if not all, methylation detectable via SMRT and MinION sequencing approaches is based on the following. First, only adenines, which are the target of the type I system, were consistently detected as methylated (m6A) in numerous GAS strains via SMRT sequencing. Second, no methylation was detected by SMRT sequencing in a GAS strain naturally lacking an intact type I RM system due to phage insertion. Finally, genetic inactivation of the type I MTase in two *emm* types dramatically decreased the methylation signal by MinION sequencing. These data are in accord with a previous study of a single *emm28* strain using SMRT sequencing in which inactivation of the type I RM system abolished genome-wide m6A methylation (25). Given the nature of predicting methylation sites using long-read sequencing data, there is not a binary answer to whether any methylation is detected, but the non-m6A methylation detected in our SMRT sequencing was inconsistent, likely indicating “noise” rather than true methylation, as has been previously described (52). Inasmuch as SMRT sequencing does not readily detect m5C methylation (53), the presumed target of type II RM systems that showed low transcript levels under the conditions studied, it remains a formal possibility that the phage-encoded, variably present type II systems do contribute to GAS methylation. The recent development of a WGS approach to detect m5C methylation could help to clarify the role of type II RM systems in GAS (53).

By performing SMRT sequencing on numerous GAS *emm* types, we deduced the target sequences of the type I system for nearly all of the publicly available, fully sequenced GAS strains in our database, with the exception of two strains that carry a rare TRD_I allele. In turn, these TRD assignments in combination with phylogenetic clustering allowed for discernment that GAS *hsdS* gene composition has likely been shaped by horizontal gene transfer (HGT) of TRD-encoding subunits. This stands in contrast to the HsdS structure observed in Staphylococus aureus, in which HsdS composition tracks with clonal complexes (54). The HsdS conservation among related S. aureus strains is thought to explain the limited exchange of genetic material between various clonal complexes (54). The GAS type I HsdS population structure seems more closely related to those of Staphylococcus epidermidis (52) and S. pneumoniae (55), in which HsdS composition does not align with whole-genome relatedness and is thought to facilitate recombination among genetically diverse strains. It is tempting to hypothesize that the presence of identical TRDs in genetically distinct GAS isolates would permit interstrain transfer of genetic material, and indeed, we identified that *emm1* and *emm12* isolates, which share nearly identical *nga-slo* regions, also have the same TRD combination (35). However, when more broadly applied to the GAS population, we did not find a clear signal that TRD composition correlated with accessory genome content, suggesting that additional factors are important for the acquisition of key GAS adaptive genes encoding superantigens and DNases. GAS also harbors CRISPR-Cas systems, another major mechanism used by bacteria to distinguish between self and nonself (56), and this might explain, in part, the incongruency observed between non-core chromosomal content and TRD distribution. Knowledge of the TRD combinations in various *emm* types could assist with the choice of vectors for genetic manipulation of GAS and even facilitate designing genetic changes to permit vector use when there are incompatibilities with particular TRD target sequences (28, 57).

Another key finding of our work was that the conserved GAS type I RM system is distinct from those of S. pneumoniae (21, 58) and S. suis (23), which contain multiple HsdS genes arranged in tandem at the 3′ end of the *hsdRSM* operon. Recombination among the *hsdS* genes results in strains with distinct methylation patterns and distinct transcriptomes, presumably through differential methylation of promoter DNA that in turn influences transcription (21, 23, 58). In contrast, the GAS *hsdRSM* operon contains a single *hsdS* gene. To date, the ability of prokaryotic type I RM systems to influence gene expression has been limited to those with the capacity to switch between various *hsdS* alleles, as seen in S. pneumoniae and S. suis (59, 60). Deletion of the type I RM system in an *emm28* GAS strain was reported to strongly reduce the expression of *mga* and Mga-regulated genes (25). However, a recent study of *emm1* GAS reported no impact of the loss of the type I system on gene expression (26). In accordance with Finn et al. (26), we found that inactivation of the type I MTase did not impact *mga* or Mga-regulated gene transcript levels in two distinct GAS *emm* types. It is well established that laboratory manipulation of GAS can result in downregulation of *mga* and Mga-regulated genes through unclear mechanisms, so it is possible that the previous observation resulted from such a phenomenon (42). Alternatively, the difference between the previous study and our findings could have resulted from strain-specific findings. The identification of clinical isolates from multiple GAS *emm* types that contain inactivation or even absence of the type I system also argues against a significant contribution of the type I RM system to GAS virulence (30). Nevertheless, the fact that vast majority of GAS strains do contain a type I RM system suggests that it is important for the overall fitness of the bacteria from an evolutionary standpoint.

We also sought to discern the origin of the type I GAS system through a comparative sequence approach. Surprisingly, streptococcal strains closely related to GAS, such as S. dysgalactiae or S. equi, did not contain clear homologs of the type I system, indicating that GAS may have acquired the system through HGT or that loss of the system has occurred in closely related streptococci. Interestingly, nearly identical type I systems were found in occasional S. agalactiae (also known as GBS) strains, which despite the nomenclature, are not closely related to GAS. A major recombination event between GBS and GAS has been identified involving *emm28* strains (36), and the type I system in GBS does contain a TRD1 allele nearly identical to that present in *emm28*. This finding raises the possibility that the presence of identical or near-identical TRD combinations in GAS and GBS might have facilitated the recombination event that seems to have been critical in GAS *emm28* strains being the major cause of puerperal sepsis, an infection typically caused by GBS (61). The scattered nature of the type I system in GBS suggests it may have been imported from GAS rather than serving as the source. Thus, at present, the origin of the GAS type I system remains obscure.

Finally, our analysis also revealed that all but 3 of the 224 GAS strains analyzed possessed orphan type II methyltransferases present in mobile genetic elements. Methylation (m5C) activity has been reported for a type II system in an *emm6* GAS strain that contains both an REase and MTase (24). However, we did not detect any m5C modifications in the strains sequenced by PacBio in this study, all of which harbor orphan type II MTases. The impact of these orphan type II MTases in GAS remains unclear since they have very low expression under the conditions studied herein.

In summary, we have characterized from a bioinformatic and biologic standpoint a type I RM system that is the lone RM system consistently found in group A Streptococcus. Unlike other streptococci, the system seems primarily involved in protection against exogenous DNA. Knowledge of the type I RM system may facilitate future efforts to genetically manipulate this important pathogen.

## MATERIALS AND METHODS

### Growth and DNA isolation, mutant construction, and GAS electroporation.

Bacterial strains listed in Table 2 were routinely grown in Todd-Hewitt (THY) broth at 37°C with 5% CO_2_. Isogenic *hsdM* mutations in MGAS6180 and TSPY1057 were obtained by nonpolar insertional mutagenesis with a spectinomycin cassette as described before (44). To determine electroporation efficiency, competent GAS cells were transformed with 1 μg of pLZ12 plasmid DNA (39) that carries a spectinomycin resistance cassette. Cells were allowed to recover for 2 h, and dilutions were plated on THY agar plates with spectinomycin (150 mg/ml) and enumerated after overnight growth. Primers are listed in Table S3 in the supplemental material.

10.1128/mSphere.00799-21.9 TABLE S3 Primers and probes used in this study. Download Table S3, XLSX file, 0.01 MB. Copyright © 2021 DebRoy et al. 2021 DebRoy et al. https://creativecommons.org/licenses/by/4.0/ This content is distributed under the terms of the Creative Commons Attribution 4.0 International license.

### Generation of reference genomes.

Complete genome sequences were determined for several GAS strains in this study using a combination of short-read (Illumina) and long-read (Oxford Nanopore) sequence data (Table 2; Table S2a). Genomic DNA extraction, library construction, and sequencing (Illumina MiSeq or ONT GridION) were performed as previously described (62, 63). Average depths of coverage for completed genomes were >100-fold and a minimum of 50-fold for both short- and long-read sequences. Hybrid genome assemblies were determined using Unicycler v0.4.6 (64) and annotated using PGAP at NCBI (65). Accession numbers for completed genomes are provided in Table S2a.

### SMRT and MinION sequencing.

GAS strains were harvested at mid-exponential phase, and high-quality genomic DNA was isolated using the Master Pure kit (Lucigen) for sequencing by the PacBio or MinION system. SMRT sequencing was performed at the Johns Hopkins Deep Sequencing and Microarray Core. For PacBio RS II sequencing, 10 to 20 libraries were prepared following the manufacturer’s recommended procedure using the PacBio SMRTBell Template Prep kit v1.0 with BluePippin size selection. Each library was sequenced using polymerase binding kit P6v2 and sequencing kit 4v2 (C4 chemistry) on one SMRTcell. The sequencing data were analyzed using PacBio smrtanalysis software v2.3.1 base modification and motif analysis pipeline.

ONT library preparation was performed using the Rapid barcoding kit (SQK-RBK004) with 400 ng of genomic DNA (gDNA) as input using the manufacturer’s protocol. A MinION (R9.4.1) flow cell was used on the GridION platform (Oxford Nanopore Technologies) with base calling and demultiplexing performed offline with Guppy v4.5.2. Flye v2.8 was used to create long-read assemblies using the ONT long-read data. In order to detect methylation using ONT data, a neural network classifier was trained using the MGAS6180 PacBio data as the “gold standard” with the mCaller software package. A 50% data set split of detected m6A motif sites identified by PacBio in the MGAS6180 genome (1,222 sites) was used for training and testing, respectively. We performed 5-fold cross validation training on the mCaller neural network model using the MGAS6180 train set and obtained a cross validation accuracy of 0.82 ± 0.03. The test set of detected m6A positions for MGAS6180 was used to determine the test data accuracy of the MGAS6180 ONT data (180/607 = 30%). The motif recognition argument of 'CRAANNNNNNNTGC' was used for methylation prediction of the 3 remaining constructs with motifs detected using their respective *de novo* Flye genome assemblies. The accuracies of MGAS6180 Δ*hsdM* (motifs predicted = 10/835 = 1.1%), TSPY1057 (604/818 = 73.8%), and TSPY1057 Δ*hsdM* (9/850 = 1.0%) were determined using a 50% predicted probability of methylation threshold with a minimum read depth of 10 reads. The prediction model is available on the mCaller GitHub page (38; https://github.com/al-mcintyre/mCaller).

### Pangenome and phylogenetic analysis.

There were 224 GAS complete and draft genomes that were included from NCBI as well as our study for the purpose of performing a pangenome analysis using Panaroo 1.2.4 (66). Gene content in >99% of the 224 GAS genomes was used as the cutoff threshold for the core genome. The gene cluster output was then aligned using Mafft v7.471 to create a multiple-sequence alignment. A maximum likelihood phylogenetic tree inferred from the core gene alignment was created using IQ-TREE multicore version 2.0.6 using ModelFinder, which selected a generalized time-reversible nucleotide substitution model with a FreeRate model for heterogeneity across sites using 3 categories. Additionally, bootstrap support for the maximum likelihood phylogenetic tree was added using an unbiased estimate with UFBoot2. The web-based iTol v6 software was used for phylogenetic tree visualization. The binary gene presence/absence matrix generated from Panaroo was used as input for PANINI to perform a t-SNE analysis to explore patterns of relatedness within the accessory genomes of GAS genomes and grouped by TRD pattern and *emm* type.

### Characterization of *hsdRSM* operon.

To identify homologs of GAS HsdM and HsdR in other prokaryotes, we performed a blastp search of the nonredundant protein sequences in the NCBI database using the GAS HsdRSM from MGAS2221 (*emm1*) while excluding GAS.

Conserved regions from the consensus sequence of a Mafft nucleotide alignment of the *hsdS* gene from 221 GAS isolates were used to create *in silico* primers to extract the TRD1 and TRD2 regions, respectively, using Cutadapt. The amino acid sequences of TRD1 and TRD2 were then subsequently aligned with Mafft, and the multiple-sequence alignment was used to create a phylogenetic tree using a neighbor-joining method with Geneious software. The determination of TRD groups (e.g., TRD_A) was accomplished using the RhierBAPS clustering algorithm tool, with each cluster assigned to a sequence and annotated on the neighbor-joining tree. TRD site motifs were identified using REBASE prediction, along with blastp results that have 100% identity and 100% coverage of the *hsdS* gene against previously characterized *hsdS* genes that are archived in REBASE, as well as blastp results against *hsdS* that have been characterized in our study using the PacBio SMRT analysis software. We were able to infer TRD site motifs from PacBio data with *hsdS* genes that clustered at >92% identity. JalView software was used to visualize the alignment of *hsdS* for 10 representative GAS genomes that had unique TRD patterns. A MUSCLE nucleotide alignment was generated from the 36-kbp *nga*-*slo* region for four isolates (MGAS5005 [*emm1*], SF370 [*emm1*], NCTC8332 [*emm12*], and MGAS9429 [*emm12*]). SNP-dists was used to convert the fasta alignment file into a single nucleotide polymorphism (SNP) distance matrix, where pairwise SNP distances were calculated. The comparison of *hsdRSM* operons between GAS and GBS isolates was performed using EasyFig (67).

### TaqMan qRT-PCR.

For qRT-PCR, samples were grown in duplicate on 2 separate days as described above. Cells were harvested at mid-exponential phase. RNA was prepared using the Qiagen RNeasy kit and processed as described earlier (68). Primers and probes used are listed in Table S3.

### Data availability.

The data that support the findings of this study will be shared upon publication. Whole genome sequences for GAS strains TSPY136 (CP060647), TSPY1309 (CP060644), TSPY416 (CP060643), and TSPY125 (CP007562.1) were obtained during this study and submitted to GenBank.
