# Supplementary figures and images for: Characterization of the Type I Restriction Modification System Broadly Conserved among Group A Streptococci
Source: mSphere. 2021 Nov 17;6(6):e00799-21. doi: 10.1128/mSphere.00799-21 (PMC8597746; doi:10.1128/mSphere.00799-21)

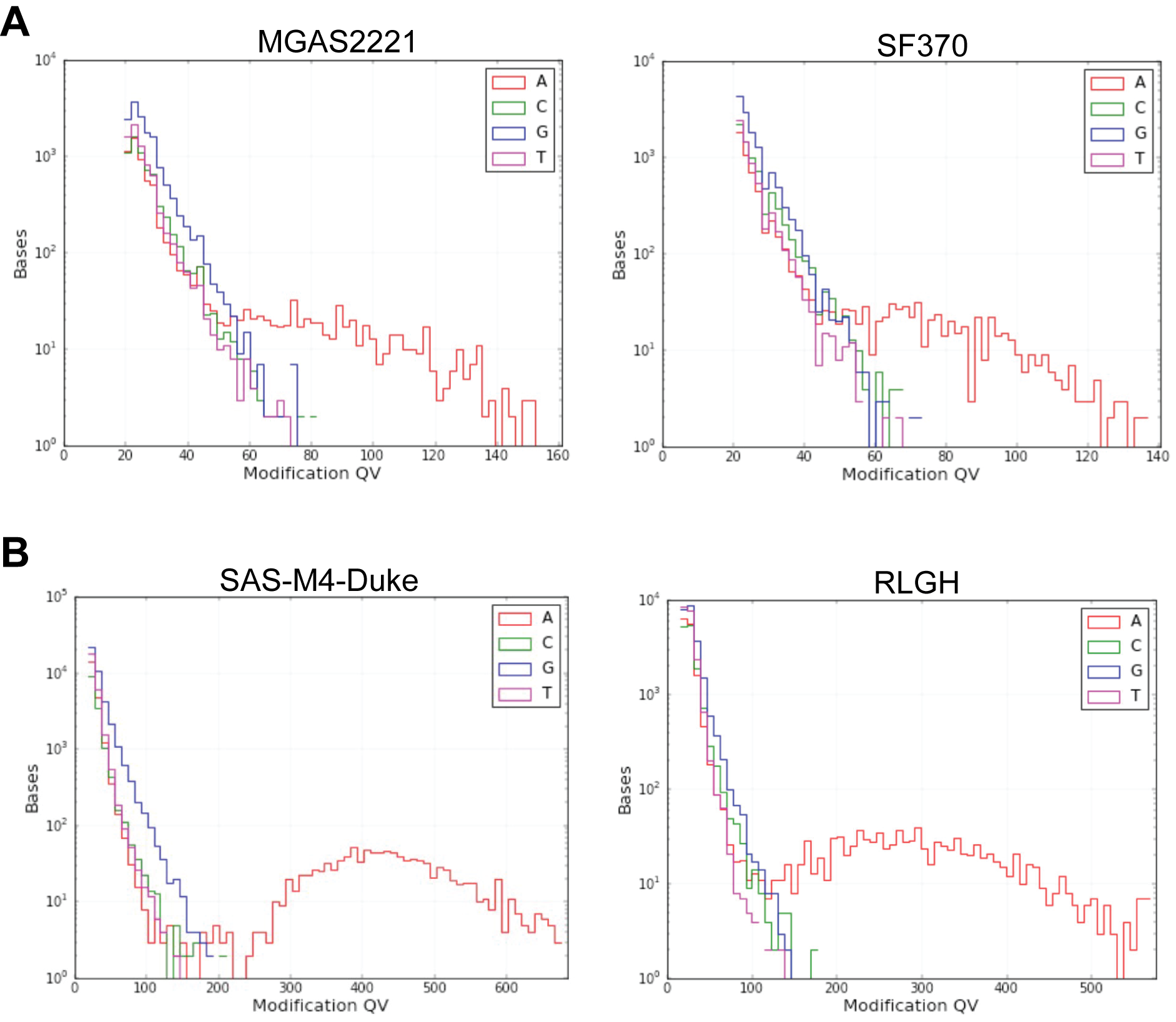

Supplement: FIG S6 [file msphere.00799-21-sf006.tif]
